# Effects of single nucleotide polymorphism ascertainment on population structure inferences

**DOI:** 10.1093/g3journal/jkab128

**Published:** 2021-04-19

**Authors:** Kotaro Dokan, Sayu Kawamura, Kosuke M Teshima

**Affiliations:** 1 Graduate School of System Life Science, Kyushu University, Fukuoka 819-0395, Japan; 2 Department of Biology, Kyushu University, Fukuoka 819-0395, Japan

**Keywords:** ascertainment bias, population structure inference, population genetics, SNP

## Abstract

Single nucleotide polymorphism (SNP) data are widely used in research on natural populations. Although they are useful, SNP genotyping data are known to contain bias, normally referred to as ascertainment bias, because they are conditioned by already confirmed variants. This bias is introduced during the genotyping process, including the selection of populations for novel SNP discovery and the number of individuals involved in the discovery panel and selection of SNP markers. It is widely recognized that ascertainment bias can cause inaccurate inferences in population genetics and several methods to address these bias issues have been proposed. However, especially in natural populations, it is not always possible to apply an ideal ascertainment scheme because natural populations tend to have complex structures and histories. In addition, it was not fully assessed if ascertainment bias has the same effect on different types of population structure. Here, we examine the effects of bias produced during the selection of population for SNP discovery and consequent SNP marker selection processes under three demographic models: the island, stepping-stone, and population split models. Results show that site frequency spectra and summary statistics contain biases that depend on the joint effect of population structure and ascertainment schemes. Additionally, population structure inferences are also affected by ascertainment bias. Based on these results, it is recommended to evaluate the validity of the ascertainment strategy prior to the actual typing process because the direction and extent of ascertainment bias vary depending on several factors.

## Introduction

Recent developments in genotyping technology have made it possible to use vast amounts of genetic information, and have drawn increasing attention to the usefulness of SNPs in ecology, evolution, and medical sciences (Brumfield *et al.* 2003; The International HapMap Consortium 2003; Manolio *et al.* 2008; Brito and Edwards 2009; Ng *et al.* 2009). In population genetics, multiple unlinked markers allow the estimation of demographic parameters and population structure inferences. SNPs are particularly effective as multi-locus markers because they can provide extensive genomic coverage, simpler mutational mechanisms than those of microsatellites, and a more reliable characterization than that obtained through AFLPs and RFLPs (Kuhner *et al.* 2000; Brumfield *et al.* 2003; Morin *et al.* 2004). However, while being convenient, SNP data contain bias, and this is an aspect researchers need to take into account.

SNPs are usually identified in a small number of samples, namely, a discovery panel. Identified SNPs are then genotyped from a large number of samples in order to collect variant data. Because SNPs found in a discovery panel represents only a fraction of the variable sites in the original population, the genotype data contain information that is to a degree ‘distorted’, as they depend on the specific original population and the number of individuals considered in the discovery panel. The existence of this ascertainment bias due to SNP selection is well known and has been investigated in previous studies (Rogers and Jorde 1996; Kuhner *et al.* 2000; Nielsen 2000; Wakeley et al. 2001; Akey *et al.* 2003; Nielsen and Signorovitch 2003; Nielsen 2004; Nielsen *et al.* 2004; Clark *et al.* 2005; Lachance and Tishkoff 2013; Quinto-Cortés et al. 2018). For example, the frequency spectrum obtained from typing data will show an excess of intermediate frequency variants because polymorphic sites are more likely to be detected when allele frequencies are intermediate. Since various summary statistics used in population genetics inferences (*e.g.*, π, Tajima’s *D*, and so on) depend on the allele frequency spectrum, this bias can cause inaccurate estimations.

To address ascertainment bias issues, methods that take into account the bias have been proposed (Wakeley *et al.* 2001; Nielsen and Signorovitch 2003; Marth *et al.* 2004; Nielsen *et al.* 2004; McGill *et al*. 2013). These approaches allow a more accurate analysis with SNP typing data by explicitly incorporating the ascertainment processes into theoretical population genetics models. However, it is not always possible to generate a discovery panel that ensures the ascertainment bias is corrected, because the choice of ascertainment schemes is often up to individual researchers (Albrechtsen *et al.* 2010). For example, various studies used typing data to elucidate the structure and history of global cattle populations (e.g. Mckay *et al.* 2008; McTavish *et al.* 2013), but drawing a detailed inference was not a straightforward process due to the existence of ascertainment bias. It is not clear if the results of these studies were comparable because the populations and criteria of the SNP loci used were ascertained differently. Moreover, it is not always possible to correct ascertainment bias because of the lack of sufficient information on the ascertainment process. Therefore, it is paramount to investigate the effects of bias on various population structure models, under various ascertainment schemes.

The effects of ascertainment bias on population structure inference have been investigated using estimates such as $F_{ST}\;$and principal component analysis (PCA) under various ascertainment schemes (Conrad *et al.* 2006; Rosenblum and Novembre 2007; McVean 2009; Albrechtsen *et al.* 2010; McTavish and Hillis 2015; Malomane *et al.* 2018). Conrad *et al.* (2006) reported that individual SNPs depend heavily on ascertainment schemes, while highly polymorphic markers are relatively unaffected by them. Rosenblum and Novembre (2007) used an empirical approach to assess the effect of ascertainment bias and found that the estimated summary statistics were very sensitive to SNP discovery strategies, highlighting the importance of the design of the initial discovery panel. McTavish and Hillis (2015) investigated the impact of ascertainment bias on population demographic inference using cattle data and showed the estimates of $F_{ST}$ and principal components can be distorted. Malomane (2018) compared SNP ascertainment schemes using genetic data from chicken, in order to elucidate and consequently reduce ascertainment bias. All of these studies were performed using empirical data and therefore they were subjected to the restrictions imposed by the actual population history. In contrast, McVean (2009), conducted theoretical population genetics studies and investigated a genealogical interpretation of PCA, showing that it can be understood as the representation of the coalescent time for samples. By considering the joint genealogy of the discovery and genotyped samples, the study also concluded that SNP ascertainment will have a simple and predictable effect on principal components. Although the influence of ascertainment bias—caused by the underrepresentation of rare SNPs in a small discovery panel—and additional bias due to uneven sampling were discussed in this study, other factors, such as threshold frequency and choice of SNP markers should also produce relevant effects. Moreover, if the population structure is different, the effects of SNP ascertainment can vary even when the ascertainment scheme is the same. Thus, it is necessary to consider different demographic histories during the evaluation of ascertainment bias.

Previous studies examined the effects of sampling location and marker selection on ascertainment bias. However, since the effects can vary based on demographic background, the combined effect of ascertainment schemes and demographic histories needs to be investigated. Here, we assess the effects of ascertainment bias under various combinations of population structure and ascertainment scheme. In this research, each ascertainment scheme involves the choice of populations to be used for the discovery samples and the methods to select SNP markers from the discovered variants. To evaluate the direction and extent of ascertainment bias, the pattern of distortion affecting the site frequency spectrum (SFS) and expected heterozygosity are analyzed. The effects on population structure inference are assessed by evaluating the variation of $F_{ST}$ and PCA results. Throughout the study, we demonstrate that the effect of ascertainment bias depends not only on ascertainment schemes but also on population structure.

## Methods

**Figure 1:**
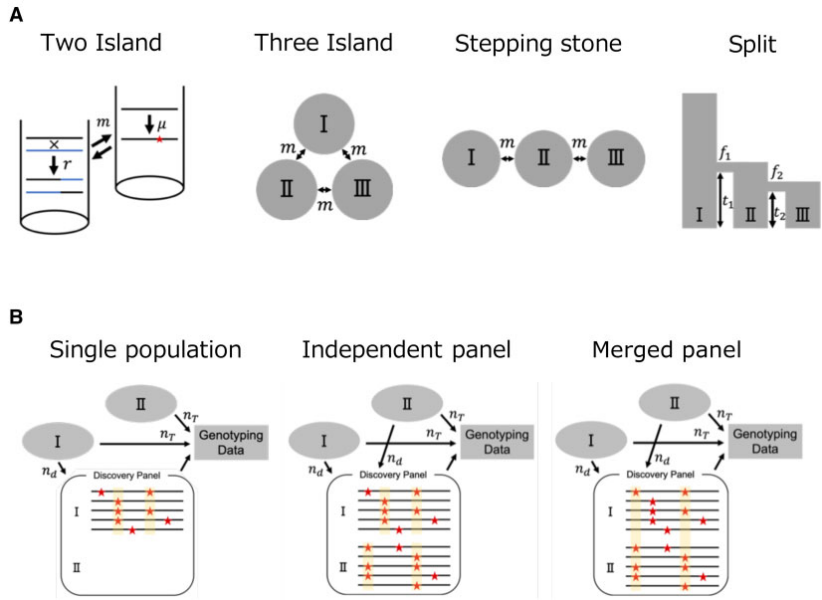
Demographic models and ascertainment schemes. Demographic histories (A) and ascertainment schemes (B) considered in the present study: (A) Island model, migration events are symmetric between populations; Stepping stone model, migration events are restricted to events occurring between adjacent populations; Split model, populations emerge from ancestral populations. (B) Single population scheme, ascertainment samples are selected from a single population. SNPs with low minor allele frequency (MAF) are removed; independent panel scheme, ascertainment samples are selected from each population, SNPs with low MAF in each panel are removed; Merged panel scheme, ascertainment samples are selected from each population, SNPs with low MAF in the merged panel are removed.

In order to investigate the combined effect of ascertainment methods and demographic history, four demographic histories and three ascertainment schemes were considered in this study (Figure 1). Details of the demographic histories and ascertainment schemes are described below.

### Demographic models and simulation

We generated typing data by conducting simulations under various population models (see Figure 1A). Initially, two- and three-island models with symmetric migration among populations were used. The migration parameter used was $4N_{0}m=0.1$, 0.3, and 0.5 in the two-island model, and $4N_{0}m=0.3$ in the three-island model, where $N_{0}$ is the population size and m is the migration rate per generation. Population size, $N_{0}$, was the same in each model and it was kept constant. The next model was a 1D stepping stone model with three populations. Migration was restricted to events occurring between adjacent populations (4Nm=0.3). Population size was the same and it was kept constant ($N_{0}$) in this model as well. The last was a population split model with three populations. The size of the population-I was constant ($N_{0}$) throughout the simulation period. Population-II was derived from population-I at $t_{1}$. The initial size of population-II was $f_{1}\times N_{0}$, where $f_{1}$ is the relative size of the founder population. The size increased to $N_{0}$ after one generation and remained constant until present. Population-III was derived from population-II at $t_{2}$. The initial size of population-III was $f_{2}\times N_{0}$, where $f_{2}$ is the relative size of the founder population. After one generation, the size increased to $N_{0}$ and remained constant. There was no migration between populations since the population split under this model. Both $t_{1}$ and $t_{2}$ were counted in units of ${4N}_{0}$ generations.

Coalescent simulations were conducted for each model and *ms* software (Hudson 2002) was used to generate genetic variation data. In each population, we simulated 100 discovery samples and 100 additional typing samples. In this study, the discovery samples were used to obtain the newly discovered variable sites and were not combined into typing samples: when the number of SNP discovery and typing samples were $n_{d}$ and $n_{T}$, respectively, $n_{d}+n_{T}$ samples were generated in the simulation. We used θ=20 and ρ=20, where $\theta =4N_{0}\mu l$, $\rho =4N_{0}rl$, μ is the mutation rate per site per generation and r is the recombination rate. These values roughly correspond to a l=50 kb region, if we assume $N_{0}=10^{4}$, $r=\mu =10^{-8}$ per site per generation. In the split model, $f_{1}=0.2$, $f_{2}=0.1$, $t_{1}=0.3,$ and $t_{2}=0.2$ were used, which are plausible values for human evolution (The International HapMap Consortium 2005; Schaffner et al. 2005; McVean 2009). The number of replications was 10,000 per parameter set. In this study, the simulation data before typing are named re-sequencing data and they were processed with in-house scripts in order to emulate genotyping processes.

### Typing data generation

The collection of typing data involved two processes: first, the SNP discovery process and, subsequently, the typing process which determines the state of each marker on each sample. It is known that these processes introduce ascertainment bias depending on how data are collected.

To examine the effect of ascertainment bias on typing data, three ascertainment schemes were considered (Figure 1B). In the first, $n_{d}=100$ discovery samples were selected from a single population (which was population-I, unless otherwise stated) and variable sites were examined. When the minor allele frequency of a variable site was lower than the predefined threshold, then the site was eliminated. Within the remaining candidate markers, 50 SNP sites were randomly selected as SNP markers. When the number of remaining candidate markers was <50, the replicate was not considered in the subsequent analysis. We named this ascertainment scheme as ‘single population scheme’. In the second ascertainment scheme, 100 samples were selected from each population, and all the SNP discovery samples were merged to build a single panel. Variable sites were detected in the panel, and those with minor allele frequencies below the threshold were filtered out. Within the remaining candidate sites, 50 were randomly selected. We named this ascertainment scheme as ‘merged panel scheme’. In the third scheme, 100 samples were selected from each population and they were used to build an independent discovery panel. Variable sites that had minor allele frequencies below the threshold in any of the three populations were eliminated. The remaining candidate sites from all panels were merged and 50 marker sites were randomly selected. We named this third ascertainment scheme as ‘independent panel scheme’. The average number of SNPs before and after the ascertainment, and the number of SNP markers are summarized in Supplementary Tables S1 and S2.

Typing data were obtained from the samples by determining the state of marker sites selected from each population. The size of the typing sample was set $n_{T}=100$ samples. The 2D-SFS and the average number of differences between sample pairs within- and between-populations (denoted by $\hat{\pi_{w}}$ and $\hat{\pi_{b}}$, respectively) were calculated. Also, the measure of the divergence between populations $\hat{F_{ST}}=1-\frac{\hat{\pi_{w}}}{\hat{\pi_{b}}}$ was calculated (Wright 1951; Hudson *et al.* 1992) and the effect on population structure inference was assessed by performing PCA using EIGENSTRAT (Patterson *et al.* 2006; Price *et al.* 2006). Typing data for PCA were generated by performing simulations using the demographic models with three populations and removing variable sites that presented a frequency of <5% in the discovery panel. All the remaining sites were used to conduct PCA.

## Results

### Effects of ascertainment bias under the two-island model

**Figure 2:**
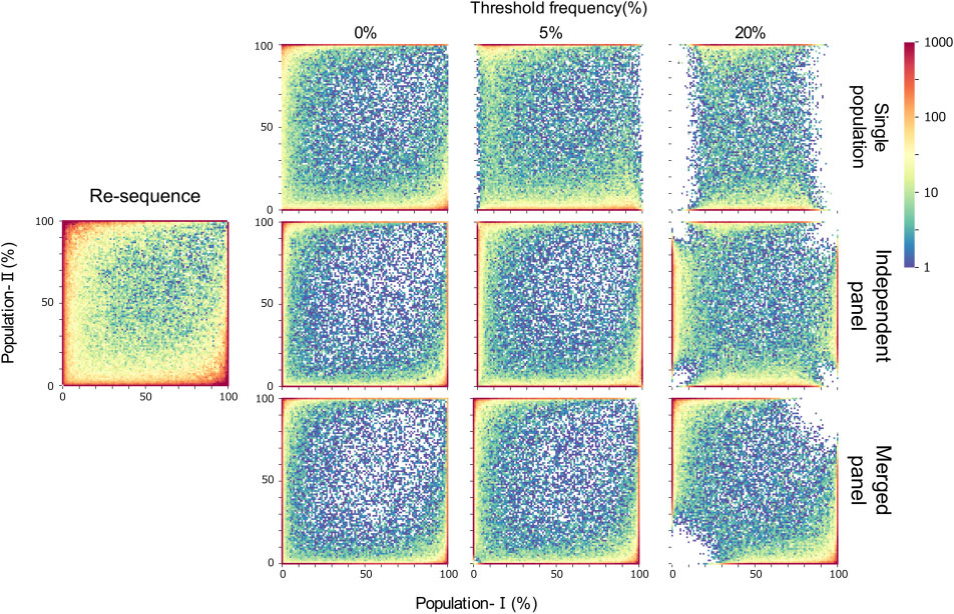
2D-SFS under different ascertainment schemes. Two-dimensional site frequency spectra of re-sequencing data and typing data for various ascertainment schemes and threshold frequencies (4Nm=0.1 was used). The x and y axes indicate derived allele frequencies in population-I and -II, respectively.

At first, coalescent simulations were conducted under the two-island model to examine the effect of ascertainment in typing data. The observed patterns for the 2D-SFS are shown in Figure 2. As confirmed by previous studies (Nielsen and Signorovitch 2003; Nielsen 2004; Clark *et al.* 2005), the observed SFS was skewed compared with the unbiased spectrum. This distortion indicates the presence of ascertainment bias, which depended on the sampling location of discovery panels and methods used for marker generation.

When markers were designed under the single population scheme, the presence of low and high frequency SNPs in population-I was reduced in typing data. The spectrum of population-II, however, was not significantly affected compared with the spectrum of population-I, because the ascertainment depended on the frequency in the other population. In other words, the 2D-SFS shows an asymmetric pattern if markers are designed on a single population, even if the population structure is symmetric. When markers were designed under the merged panel or independent panel schemes, the 2D-SFS showed a symmetric pattern in the populations, because the ascertainment process reflected the variant-related information of both populations. Under the merged panel scheme, variants with rare minor alleles present in the entire population were eliminated from the obtained typing data. On the other hand, if markers were selected under the independent panel scheme, variants presenting rare minor alleles, both in the entire population and in at least one of the two populations, were removed. These 2D-SFS patterns became noticeable as the threshold frequency of marker selection increased.

**Figure 3:**
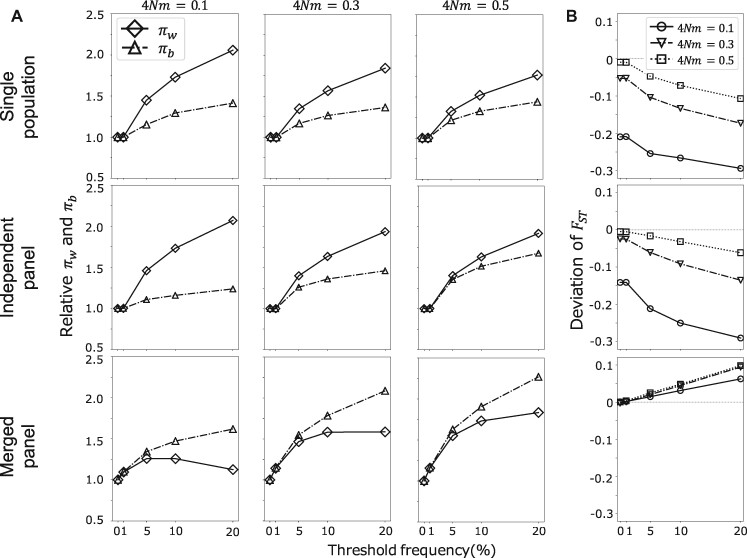
$\hat{\pi_{w}}$ , $\hat{\pi_{b}}$, and $\hat{F_{ST}}$ under different ascertainment schemes. (A) Variation of $\hat{\pi_{w}}$ and $\hat{\pi_{b}}$ for ascertainment schemes and the degree of migration between populations. x and y axes indicate the threshold frequency of marker selection and relative variations, respectively. (B) $\hat{F_{ST}}$ deviation for ascertainment schemes. x and y axes indicate the threshold frequency of marker selection and $\hat{F_{ST}}$ deviation from the estimate using re-sequencing data, respectively.

Next, we considered the amount of variation in typing data, specifically, in terms of the differences between a pair of samples randomly selected from the same population, $\hat{\pi_{w}}$, and different populations, $\hat{\pi_{b}}$. It is worth noting that the amount of within- and between-population variation here considered should not be interpreted as an estimate of diversity or divergence, because the variation was calculated from typing data. The purpose of this analysis was to rather investigate the pattern of within- and between-population variation, under the influence of marker selection. In relation to this, values that are relative to those calculated using randomly selected variants are plotted in Figure 3A, where it is shown that $\hat{\pi_{w}}$ and $\hat{\pi_{b}}$ calculated using typing data were generally overestimated. As previously described, genotyping procedures involve the removal of loci with rare minor alleles, which is equivalent to an increase of intermediate frequency SNPs in the proportion. The vertical axes of Figure 3A indicate the amount of variation calculated based on randomly chosen markers, and here it increases as the threshold frequency of marker selection increases, regardless of the ascertainment scheme employed. The incremental pattern, however, differed among schemes: the increment of $\hat{\pi_{b}}$ was larger than that of $\hat{\pi_{w}}$ when the merged panel scheme was applied, whereas the increment of $\hat{\pi_{w}}$ was always larger than $\hat{\pi_{b}}$ in the other schemes. This difference can be explained by the 2D-SFS pattern shown in Figure 2.

Under the merged panel scheme, SNPs were eliminated only if the minor allele was rare in both populations, and this selectivity contributed to the increase of heterozygosity within populations. When SNP markers were generated using the other schemes, variants with rare minor alleles in one of the two populations were also removed, in addition to those carrying rare minor alleles in both populations. In other words, diverged SNPs between two populations were eliminated from the data under the single population and independent schemes. The reduction of diverged SNPs suppresses divergence and is reflected in the lower increment of between-population variation rather than that of within-population variation.

The extent of the effect of ascertainment also depends on the degree of divergence between populations. Patterns of variation under differently diverged populations are plotted in Figure 3A. The difference between $\hat{\pi_{w}}$ and $\hat{\pi_{b}}$ is larger in more diverged populations (4Nm=0.1) than in less diverged populations (4Nm=0.3 and 0.5). There are more differentiated variant sites in diverged populations than in less diverged populations (Supplementary Figure S1, top row). Once the ascertainment scheme is applied, variants with rare minor alleles in population-I are excluded from the analysis (Supplementary Figure S1, bottom row). Since highly differentiated variants are eliminated, the deviation of the SFS from the original distribution in diverged populations increases compared with the deviation in less diverged populations. When the divergence between populations is small, due to the effect of migration, bias is relatively limited because most variable sites are shared between populations in similar frequencies. These observations also mean that the effect of bias is stronger on $\hat{\pi_{b}}$ than on $\hat{\pi_{w}}$, because differentiated sites with rare minor alleles are eliminated (Supplementary Figure S2). Therefore, the greater the divergence between populations is, the greater the bias difference between $\hat{\pi_{w}}$ and $\hat{\pi_{b}}$.

We have illustrated how the typing process differentially affects $\hat{\pi_{w}}$ and $\hat{\pi_{b}}$ implying that population differentiation parameters, such as $F_{ST}$, should also be affected depending on the typing scheme used. Thus, the values of $\hat{F_{ST}}$ were calculated based on the typing data, and the deviations between the observed $\hat{F_{ST}}$ of typing data and re-sequencing data were subsequently plotted (Figure 3B). The $\hat{F_{ST}}$ values of re-sequencing data were 0.806, 0.599 and 0.478 when 4Nm=0.1, 0.3, and 0.5, respectively. As shown in Figure 3B, the deviation increased as the threshold frequency of marker selection increased.

The direction of the deviation depends on the ascertainment scheme used. When the single population and independent panel schemes were applied, $\hat{F_{ST}}$ suffered downward bias. As previously described, data generated using these two schemes lost diverged SNPs and showed relatively low divergence compared with within-population variation, therefore the low $\hat{F_{ST}}$ values observed. The degree of deviation was correlated with the divergence between populations. When the merged panel scheme was applied, diverged SNPs were retained in the typed dataset, whereas SNPs that presented low or high frequencies in both populations were eliminated. That is why the observed $\hat{F_{ST}}$ values were overestimated under the merged panel scheme.

Figure 3 shows that the direction of the deviation depends on the ascertainment scheme in the two-island model. To examine if this observation changes depending on the demographic model, comparisons were carried out between the two-island and population-split models. The migration rate and split time were calibrated to obtain the same $F_{ST}$. Although the degrees of deviation were slightly different, the direction of bias was the same in the two demographic models (Supplementary Figure S3), suggesting that the choice of the ascertainment scheme plays an important role in determining the bias, regardless of the demographic model used.

### Effects of ascertainment bias under demographic models with three populations

**Figure 4:**
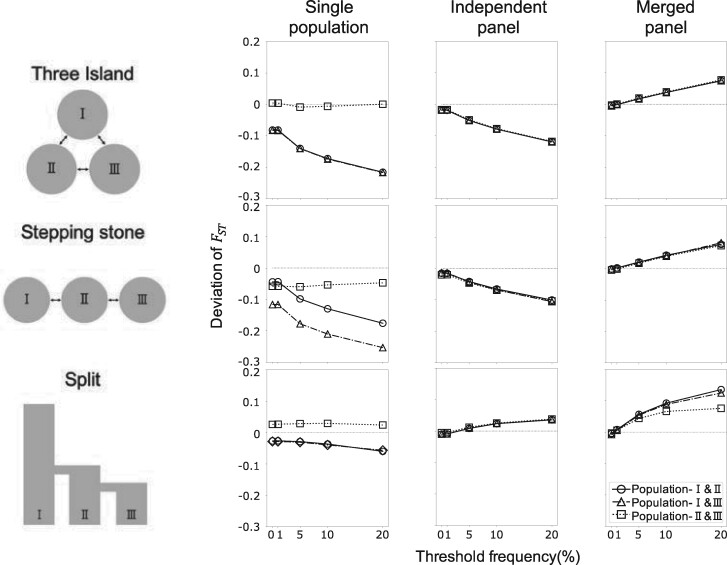
$\hat{F_{ST}}$ under the combined effect of ascertainment schemes and population demography. The combined effect of ascertainment schemes and population demography on $\hat{F_{ST}}$ estimation. Deviations of $\hat{F_{ST}}$ from the estimates with re-sequencing data are plotted against the threshold of marker selection in each ascertainment scheme. $\hat{F_{ST}}$ deviations between population-I and -II, -I and -III, and -II and -III are indicated by circles, triangles, and squares, respectively. The average $\hat{F_{ST}}$ values calculated from the re-sequencing data for each demographic model are summarized in Supplementary Table S3.

In this section, the effects of ascertainment were investigated for three demographic models with three subpopulations (Figures 1 and 4). The first model considered was an island model with symmetrical migration (three-island model). As symmetric migration was assumed, the divergence was also symmetric in this model. The second model used was a stepping stone model. Here, the divergence between populations depended on the arrangement of populations, as migration was restricted to events occurring between adjacent populations. The last model used was a population split model. In this model, the divergence depended on the order and timing of population splits. SNP discovery samples were selected from population-I in the single population scheme. In the other two schemes, the samples were selected from all three populations. Pairwise $\hat{F_{ST}}$ between populations was calculated and deviations from the unbiased estimates with re-sequencing data are shown in Figure 4.

Under the single population scheme, the direction of $\hat{F_{ST}}$ biases was similar to that observed in the two-island model, as long as population-I was considered in the analysis of population pairs. The patterns of the observed $\hat{F_{ST}}$ of population pairs for population-I showed a tendency toward underestimation, especially when variants with rare minor allele were removed. The elimination of these from population-I led to an increase in the within-population diversity, and therefore, to an underestimated $\hat{F_{ST}}$.

As shown in Figure 4, the $\hat{F_{ST}}$ between population-I and other populations was underestimated whereas $\hat{F_{ST}}$ between population-II and -III was not. This result reflects the effects of the ascertainment process under the single population scheme. Since markers were selected based on their frequency in the discovery panel originated from population-I, the typing data of population-I were more significantly affected by the ascertainment process than those of the other populations. In contrast, the typing data of population-II and -III, were not affected by their own frequencies but by the frequency of population-I. Thus, the distortion of the frequency spectrum was rather moderate. These different influences of the single population scheme on each population produced differently biased typing data. A comparison of the 2D-SFS, before and after ascertainment, will contribute to understanding how ascertainment produces different effects (Supplementary Figure S4). The 2D-SFS between population-I and other populations was distinctly different before and after the ascertainment because variants were ascertained only in population-I, whereas the 2 D-SFS between population-II and -III remained largely unaltered. Therefore, the $\hat{F_{ST}}$ of population pairs with population-I showed a clear underestimation compared with that between population-II and -III.

In the stepping stone model, the $\hat{F_{ST}}$ between population-I and -III was largely underestimated compared with the $\hat{F_{ST}}$ between population-I and -II. The divergence between population pairs in this model reflects the arrangement of populations and it was larger between population-I and -III than between the other pairs. As shown in Figure 3B, the effects of ascertainment bias on $\hat{F_{ST}}$ were pronounced on the diverged population pair. Therefore, the largest effect was observed between population-I and -III, and it was an underestimation. If the discovery samples were selected from population-II, the $\hat{F_{ST}}$ of population pairs (including population-II) was equally underestimated, whereas the $\hat{F_{ST}}$ between population-I and -III was not significantly affected by bias (Supplementary Figure S5). Thus, in order to understand the effect of ascertainment within the single population scheme, it is important to be aware of the population from which the discovery panel is selected and of the between-population distance from the ascertained population. In contrast, under the merged and independent panel schemes, the degree of bias remained unchanged among population pairs, because the discovery samples were selected from all populations.

The effects of ascertainment bias on $\hat{F_{ST}}$ under the split model appeared different compared with those observed under the other two models. The typing within the single population scheme produced little bias regardless of population pairs under the split model considered here. The $\hat{F_{ST}}$ between un-ascertained populations showed little overestimation, whereas the $\hat{F_{ST}}$ between ascertained populations showed a slight underestimation. The same results were obtained when the discovery panel was sampled from population-II or -III (Supplementary Figure S5). Tendencies toward upward bias were observed in the other models. When the independent panel scheme was used under the split model, upward biases were observed, whereas downward biases were observed under the other models. These unique aspects of the split model are attributed to the source of variation in each population. From the model assumption in this study, population-II was originated from a relatively small number of founders belonging to population-I and it then expanded its population size. Population-III similarly derived from population-II. New mutations after the population split were rare, since the establishment of these populations was rather recent. Because of their population history, most variations observed in population-III derived from population-II, and most variants in population-II were originated from population-I. Therefore, populations under the split model here considered shared variants in similar frequencies, and presented less diverged variants than populations under the other models did (Supplementary Figure S6). As observed in the two-island model, a key reason of $\hat{F_{ST}}$ underestimation was the elimination of diverged variants. This is why populations under the split model were not significantly affected by this bias.

It should be noted that the above patterns were observed under the split model, which is a plausible model for human evolution (The International HapMap Consortium 2005; Schaffner et al. 2005; McVean 2009). In general, bias effects differ depending on the degree of divergence between populations. In the split model, populations accumulate diverged variants with time, after the population splits, whereas, in the island model, some variants are shared through migration (Supplementary Figure S7). Thus, there are more diverged variants in the split model than in the island model, indicating that the split model is more susceptible to bias than the island model is, in case of equal divergence between populations (Supplementary Figure S8). The differences in terms of bias effects become more pronounced as the divergence between populations increases.

### Population structure inference with typing data

**Figure 5:**
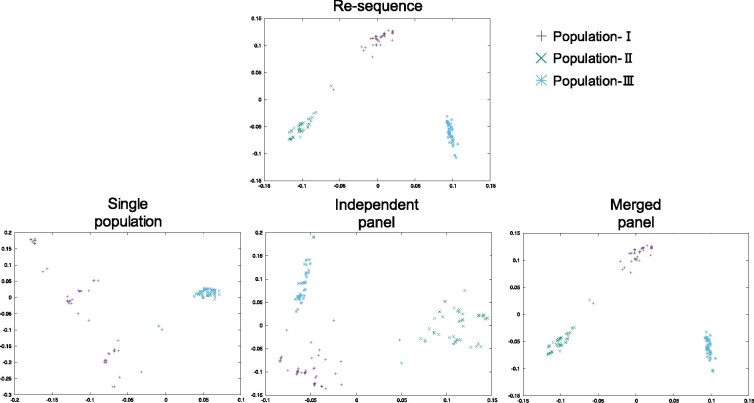
PCA performed on the simulation data under the three-island model with 4Nm=0.3. The first and second principal components are plotted based on 100 samples selected from each population. The threshold of marker selection was set as 5% and all markers were included in the analysis.

Typing data may contain distorted information depending on the typing scheme and the demographic model used. To evaluate if the ascertainment process has also an influence on population structure inference, PCA analyses were conducted under the three-island model using both re-sequencing data (no ascertainment) and typing data, by applying the three typing schemes discussed above. Results are plotted in Figure 5.

The resulting PCA patterns depended on typing schemes: distinct clusters of samples for each population were observed when re-sequencing data were used, while, when typing data within the single population scheme were used the result of PCA was different. Samples taken from population-I were widely distributed, while population-II and -III formed a single dense cluster. This pattern can be attributed to the asymmetric design of the typing scheme. Since the markers were designed based on the discovery samples from population-I, specific variants of population-II and -III were lost. As a result, the data did not possess the ability to correctly distinguish population-II and -III. Although the effects of ascertainment under the independent and merged panel schemes were symmetric, PCA results were different. If the merged scheme was used, the clustering pattern was comparable to that obtained using re-sequencing data. Since only SNPs with rare minor alleles across the entire population were eliminated, and the effects of ascertainment bias were symmetric under this scheme, the resultant structure did not present a large number of distortions. Although the bias effect was also symmetric in the independent scheme, the clusters of samples were difficult to interpret compared with those obtained using the merged panel scheme; and as population-specific diverged variants were eliminated under the independent scheme (Figure 2), some divergent information was lost in this scheme.

Based on these results, two considerations are essential to further understand the effect of ascertainment bias on population structure inference: one is whether variants in all populations are equally ascertained or not; if variants in only one population are ascertained as in the single population scheme, the inference will be inevitably skewed. The second point is which variant components are ascertained: when diverged variants are excluded from a set of SNP markers, as in the independent panel scheme, the divergence among populations will be proportionally underrepresented. In summary, for appropriate inference, it is necessary to be aware of ‘where’ discovery panels originate from and ‘which’ SNPs are selected for typing.

## Discussion

Both the choice of the SNP discovery panels and the strategy for SNP marker selection affect the influence of ascertainment bias. The impact of ascertainment bias can be understood from the analysis of the 2D-SFS pattern. When a discovery panel was sampled from a single population, the observed typing data were affected by the polymorphism of the samples in the discovery panel resulting in an asymmetric 2D-SFS (Nielsen and Signorovitch 2003; Clark *et al.* 2005; Albrechtsen *et al.* 2010). The SFS of the population used for discovery panel selection showed a deficiency of SNPs with rare minor alleles under the single population scheme, which generally leads to the overestimation of heterozygosity (Lachance and Tishkoff 2013). Since the ascertainment was performed on a single population, the effect of ascertainment bias appeared asymmetrical among populations (Nielsen 2004). In contrast, when discovery samples were selected from different populations, the variants observed in each population were ascertained. Thus, the ascertainment process has equivalent effects among populations. SNP marker selection and choice of panels both determine the direction and extent of the bias. Since population inference derived from typing data is inevitably affected by ascertainment bias, ascertainment schemes and their impacts on typing data should be well recognized prior to analysis.

It should be noted that the samples of discovery panels were not used as the typing samples in this study. However, in some previous studies, they were used to generate typing data (Wakeley *et al.* 2001; Nielsen 2004; Nielsen *et al.* 2004). Once SNPs were selected, those containing rare minor alleles were eliminated from the discovered variants (Nielsen 2004). If an extreme example is considered—where exactly the same samples are used both in the discovery and the typing panels—the observed SFS will be sharply truncated on both ends of the distribution, because variants with rare minor alleles are eliminated during the SNP selection process. When this is the case, the SFS generated from the typing data will show a large deviation from the true distribution. Therefore, when discovery samples are used in the subsequent genotyping process, the effect of ascertainment bias on population inference will be slightly more pronounced compared with cases in which the discovery and typing samples are kept separate (Supplementary Figure S9).

Divergence from the population for discovery panels also affects the extent of the ascertainment bias. In general, biases in summary statistics increase as populations diverge (Lachance and Tishkoff 2013) suggesting that distortion levels in typing data are not uniform and depend on the spatial arrangement of populations. For example, if the discovery panel were generated from a single local population, biases in the estimates would be emphasized as the distance from the population for discovery panels increases. When this is the case, the comparison of summary statistics among populations and population structure inference will become questionable.

The impact of ascertainment bias also depends on demographic histories. Ascertainment bias can be considered as the deviation of the SFS in typing data from that in the original population. Since the outcome of the ascertainment process is determined by the combination of the true SFS of populations and the applied ascertainment scheme, the efficacy of ascertainment can differ depending on population histories as well. In fact, in this study, the degree of $\hat{F_{ST}}$ deviation from the true value was slightly different between the two-island model and the split model, even when these models had the same true $F_{ST}$ or $\hat{\pi_{b}}$ (Supplementary Figures S3 and S8). This difference in the bias level can be attributed to the variation in terms of the amount of shared rare variants between the two models. Variants are more likely to be shared between populations in the two-island model than in the split model. Since diverged variants were more likely to be affected by the ascertainment process, the deviation of $\hat{F_{ST}}$ grew larger in the split model than in the two-island model. Therefore, predictions of ascertainment bias under simple population models are not necessarily applicable to interpret actual data characterized by complex population histories. Particular attention should be paid when natural populations of non-model species are analyzed.

Overall, the simulation results of this study can be summarized as follows: when the single population scheme is applied, $\hat{F_{ST}}$ is underestimated regardless of the demographic model, if a population used for the discovery panels was included in the calculations. This is attributed to the elimination of variants with rare minor allele in that population. The $\hat{F_{ST}}$ between populations, excluding the discovery population, depends on demography; although the effect of bias is generally negligibly smaller than that observed when a population for the discovery panel is included. When the independent panel scheme is applied, $\hat{F_{ST}}$ is underestimated under the three-island and stepping-stone models. As variants with rare minor alleles are eliminated in the independent scheme, population-specific diverged variants are all excluded from typing data, resulting in an underestimated $\hat{F_{ST}}$. Under the split model, the effect caused by the independent panel scheme depends on when the population split occurs, and $\hat{F_{ST}}$ is slightly overestimated when the split time is recent (Figure 4). Because of the effect of genetic drift, populations accumulate differentiated SNPs as the divergence time increases, and as these are filtered out from typing data by the effect of the independent scheme, $\hat{F_{ST}}$ eventually results underestimated, depending on the divergence time (data not shown). When the merged panel is applied, $\hat{F_{ST}}$ is uniformly overestimated, regardless of demography because population-specific differentiated SNPs are maintained under this scheme.

Different demographic histories generate different 2D-SFS patterns. When the 2D-SFS contains a certain amount of diverged variants that are not typed (as in the single population and independent panel schemes), $\hat{F_{ST}}$ will be underestimated. If diverged variants are included and variants with rare alleles in the overall population are excluded from the typing markers (as in the merged panel scheme), $\hat{F_{ST}}$ will be overestimated. Thus it is inferred that demography is one of primary factors determining the impact of ascertain bias, because it controls the 2D-SFS pattern.

Albrechtsen *et al.* (2010) pointed out that $\hat{F_{ST}}$ is less affected by ascertainment bias than other summary statistics are, because both its numerator and denominator are affected in a similar way. By considering the joint genealogy of the discovery and typing samples, McVean (2009) argued that ascertainment bias has simple and predictable effects on PCA, adding that uneven sampling has a strong influence on PCA (see also Novembre and Stephens 2008). Our findings seem to confirm this consideration, as population structure inference was found to be distorted depending on population histories and ascertainment schemes. To prevent such distortions it is essential to consider a number of aspects: (1) the discovery panels should cover the entire population otherwise estimates of summary statistics will be biased, depending on the genetic divergence form the discovery population. (2) The strategy for SNP selection from variants identified in discovery panels also affects the outcome of typing data. If SNPs are selected in each population, differentiated variants will be eliminated from the analysis, and in this case, the detection of population structures will be more difficult. As a consequence, a hidden structure may lead to misinterpretation. Thus, it is recommended to select SNPs from a collection of pooled variants, as in the merged panel scheme. It should be noted that these conclusions were derived from the simulations conducted in this research, and that the direction of ascertainment bias may vary if the population histories of samples were completely different from those considered in the present study. Even when demographic models are similar, ascertainment bias can produce different effects if summary statistics are different. Careful consideration is required since ascertainment bias derives from the combined effect of sampling strategy, data manipulation and evolutionary factors.

## Data availability

The simulation scripts—and related tutorials—necessary to replicate the analyses and confirm the results of this study can be found at https://github.com/kmteshima/SNPascertn_on_demography.git.

Supplementary material is available at https://doi.org/10.25387/g3.14374643.

## Funding

This work was supported by JSPS KAKENHI Grant Number JPS25840129 and JP19K06782.

## Conflicts of interest

The authors declare that there is no conflict of interest.
